# Instant Formulation of Inhalable Beclomethasone Dipropionate—Gamma-Cyclodextrin Composite Particles Produced Using Supercritical Assisted Atomization

**DOI:** 10.3390/pharmaceutics15061741

**Published:** 2023-06-15

**Authors:** Hsien-Tsung Wu, Han-Cyuan Lin, Yi-Jia Tu, Kim Hoong Ng

**Affiliations:** Department of Chemical Engineering, Ming Chi University of Technology, 84 Gungjuan Rd., Taishan Dist., New Taipei City 24301, Taiwan; m08138104@mail2.mcut.edu.tw (H.-C.L.); m09138206@mail2.mcut.edu.tw (Y.-J.T.); kimhoong.ng@mail.mcut.edu.tw (K.H.N.)

**Keywords:** supercritical assisted atomization, gamma-cyclodextrin, beclomethasone dipropionate, in vitro dissolution, in vitro aerosolization

## Abstract

Medical composites derived from Gamma-cyclodextrin (*γ*-CD) and beclomethasone dipropionate−gamma-cyclodextrin (BDP−*γ*-CD) are synthesized over supercritical-assisted atomization (SAA) herein. Carbon dioxide, which serves the dual function of spraying medium and co-solute, is incorporated in this process along with the ethanolic solvent. Results indicate that, for fine spherical particles, optimized aerosol performance could be obtained with 50.0% (*w*/*w*) ethanolic solvent, precipitator, and saturator at 373.2 K and 353.2 K, respectively, and carbon dioxide-to-*γ*-CD flow ratio of 1.8 in the presence of 10 wt% leucine (LEU) as dispersion enhancer. It is also noted that *γ*-CD solution at low concentration typically renders better aerosol performance of the particles. During drug particle-derivation, the solubility of drug BDP elevated considerably due to the formation of inclusion complexes, further assisted by the ethanolic solvent which increases the lipophilicity of BDP. Meanwhile, the in vitro aerosolization and dissolution performance of drug composites derived from varied *γ*-CD-to-BDP mass ratio (*Z*) were also evaluated. It was found that high *Z* promises higher fine particle fraction in the obtained drug composite while the dissolution rate of active ingredient (BDP) exhibits positive correlation to the content of water-soluble excipient (*γ*-CD) in the formulation. This study offers a new avenue for instant drug formulation with promising pulmonary delivery over the SAA technique.

## 1. Introduction

Beclomethasone dipropionate (BDP) is widely used as a glucocorticosteroid drug for respiratory and asthmatic diseases; however, its application is often shadowed by its low water-solubility (<1 μg/mL). Therefore, pulmonary delivery of such material in the form of nasal spray or dry powder inhalation is generally preferred, ascribed to the superior capability compared with other administrative approaches. Three type of inhaler devices are normally employed to facilitate the pulmonary delivery of such drug, namely nebulizers, pressurized metered dose inhalers (pMDIs), and dry powder inhalers (DPIs). Among these, DPIs are widely adopted for their propellant-free attribute, high portability and convenience, high-dose delivery, and excellent chemical and particle properties in the solid state. Water-soluble *γ*-CD is generally regarded as safe (GRAS) by the FDA and is commonly used in oral and dermal medicinal formulations [1] to improve the solubility of the active ingredient, thereby promoting the pulmonary drug delivery and bioavailability [2,3]. *γ*-CD has also been found to be low in toxicity and safe for human airway epithelial Calu-3 cells in vitro application [4]. These promise excellent potential of *γ*-CD for medical application, particularly for inhalable drugs in the form of dry powder.

Conventional inhalable dry powders are often formulated by mixing active ingredient with coarse carrier particles, where the latter function as flow and aerosolization enhancers during administration. Although such formulations provide precise drug dosages, the detachment of fine drug particles from the carrier may induce unsatisfactory delivery of the active ingredient to the lungs. Notably, the aerodynamic attributes of BDP/*γ*-cyclodextrin composite on three different carriers, namely lactose, trehalose, and respitose, were examined in the past [5], and it was confirmed that the former two had better aerodynamic performances. Meanwhile, the combination of active pharmaceutical ingredients (API) with different functional excipients has been reported to improve the physical and aerosolization properties, despite the absence of coarse or fine carriers. Meanwhile, the formulation with HP-*β*-CD and _L_-leucine (LEU) as solubilizer and coating agent, respectively, could also enhance the dissolution and aerosol performance of the obtained drug [6]. LEU, a protein-based amino acid, is widely used as a dietary additive to promote endogenous insulin release, inhibit muscle protein breakdown, and stimulate muscle protein synthesis under both healthy and diseased conditions [7]. By utilizing the spray drying approach, the deposition of LEU on the target carrier could impart a positive interfacial activity, thereby advocating for the overall aerosolization and aerodynamic nature of the resultant composite [8]. For inhalable drug synthesis, spray drying is one of the commonly employed approaches to derive dry powder from API and functional excipient mixtures. Representatively, Mohtar et al. [9] produced drug complex that consisted of sulfobutylether-*β*-cyclodextrin (SBE-*β*-CD) and fisetin using this mentioned approach, where the presence of LEU in the formulation improves drug solubility, enabling accurate delivery of a high fisetin dosage to the deep lung region. Similarly, the study by Suzuki et al. [10] develop roflumilast dry powder by dissolving HP-*β*-CD in ethanol prior to spray drying. Significantly, our previous study focused on the preparation of BDP−HP-*β*-CD composites using a supercritical assisted atomization (SAA) technique for the purpose of immediate drug release [11]. In the formulation, HP-*β*-CD functions as a carrier or solubilizer, and LEU enhances the homogeneity of the API. Given that such SAA-prepared formulations permit precise drug delivery, a BDP/*γ*-CD composite prepared using the same technique for pulmonary delivery should be equally promising; however, this has not yet been explored.

For medicine formulation, SAA is more prevailed than the conventional micronization for its high-yield operation, convenient scalability [12,13], and compatibility with thermosensitive proteins or low-melting materials [14,15,16]. Considering these advantages, this study aimed to develop a novel instant DPI formulation consisting of BDP/*γ*-CD/LEU composite particles with SAA. Post-synthesis analysis indicates an improved solubility of BDP in the DPI, which was ascribed to the formation of an inclusion complex with *γ*-CD. This, together with LEU-assisted homogenization, improved the aerosolization performance of the resultant inhalable dry powder, as evidenced by in vitro evaluation using an Andersen cascade impactor (ACI). In parallel to this, the dissolution performance of the BDP/*γ*-CD/LEU composites was augmented based on the results obtained from the dissolution tester. Meanwhile, the crystallinity and structural properties of the obtained composite particles were evaluated using X-ray diffraction (XRD) and Fourier transform infrared spectroscopy (FTIR), respectively, while the thermal behavior was examined using differential scanning calorimetry (DSC).

## 2. Materials and Methods

### 2.1. Materials

Analytical-grade gamma-cyclodextrin (*γ*-CD, 99.9% purity) and l-leucine (99.9% purity) were purchased from Sigma-Aldrich, St. Louis, MO, USA. High performance liquid chromatography (HPLC) grade ethanol and methanol were procured from Acros, Branchburg, NJ, USA. The targeted active ingredient, beclomethasone dipropionate (BDP, 99% purity), was provided by Tokyo Chemical Industry, Tokyo, Japan. Carbon dioxide (99.9% purity) and nitrogen were obtained from Yung-Ping Gas Co., Taipei, Taiwan. A high purity of 99.9% was required for both the experimental gases. All chemicals were used as-received. Deionized water (from Millipore Milli-Q) with resistivity of 18 MΩ⋅cm at 25 °C was used for solution preparation.

### 2.2. Synthesis of Drug–γ-CD Composite Particles

The schematic diagram of our SAA equipment, along with its operational procedure, were presented elsewhere, referenced herein [17]. The major chambers in the SAA, namely saturator, precipitator, and cold trap, were set in place, along with separate feeding lines for drug–*γ*-CD solution, CO_2_, and N_2_, and were connected to the setup. The heated N_2_ flow rate was 1.0 Nm^3^/h. Prior to the precipitation process, CO_2_ at preset rate was channeled into the saturator to establish a steady state. The temperature of the saturator (*Ts*) was set accordingly. Thereafter, a pre-heated drug–*γ*-CD solution stream was pumped into the saturator to absorb the coexisting CO_2_. The resulting solution was atomized by forcing through an injection nozzle in the connecting precipitator. Evaporation of the solvent was prompted simultaneously upon contact with the liquid and heated N_2_, resulting in precipitated drug–*γ*-CD composites owing to supersaturation effects.

The obtained solid samples were collected and subjected to particle size distribution (PSD) determination using a dynamic light scattering (DLS) particle analyzer (Zatasizer Nano ZS90, Malvern, UK). Significantly, well-separated solid particles are necessary for accurate DLS measurement; therefore, in the current study, the sample was pre-sonicated in petroleum oil at 298 K to establish better suspension [18]. The arithmetic and mass-weighted mean particle diameters, *d_no_* and *d*_4,3_, respectively, were calculated using the equations $d_{no}=\sum_{i=1}^{i}x_{i}D_{i}$ and $d_{4,3}=\sum_{i=1}^{i}(x_{i}D_{i}^{4}/\sum_{i=1}^{i}x_{i}D_{i}^{3})$, where *x* denotes the number fraction of the particles. All precipitation experiments were triplicated, and the average solid yield from SAA was determined to be 80%, where the losses arose from the adherence of the microparticles to the inner surface of the SAA machine, such as precipitator walls or filter pores.

### 2.3. Solid-State Characterization

The morphology of the solid particles was observed using field-emission scanning electron microscopy (FESEM, model 6500, JEOL, Tokyo, Japan). The crystallinity of selected samples was also examined using X-ray diffraction (XRD) provided by X’Pert Pro X-ray powder diffractometer (PANalytical, Almelo, The Netherlands), where the scanning was performed at a 2θ range of 5° to 50° at a rate of 0.02°/s. The surface functional groups of samples were spectroscopically identified over Fourier transform infrared spectrophotometer manufactured by Thermo Scientific Nicolet iS5 FTIR Spectrometer (Thermo Fisher Scientific Inc., Waltham, MA, USA). Attenuated total reflection (ATR) element was employed herein to enable facile FTIR examination. Meanwhile, a thermogravimetric analyzer (TGA, SDT Q600, TA, New Castle, DE, USA) was used to determine the LEU content in the *γ*-CD carrier particles produced by SAA by measuring the weight loss from room temperature to 673 K (10 K/min) under N_2_-blanket (50 mL/min). Thermograms of the composite samples in the range of 298.2–553.2 K were recorded under N_2_ atmosphere as well, using a differential scanning calorimeter (LT-DSC) from Netzsch 204 Fl Phoenix, Selb, Germany. 

Further, the bulk density (*ρ_buk_*) and tapped density (*ρ_tap_*) of the drug–*γ*-CD powder were determined and correlated to the powder cohesiveness and flow properties. For *ρ_bulk_* determination, the bulk volume of an accurately weighed drug–*γ*-CD powder was measured using a 5 mL cylinder. Next, the cylinder was programmed to 1250 times of tapping using automated tap density analyzer (Auto top 02106-60-1, Quantachrome, Boynton Beach, FL, USA) as per the recommendations of the European Pharmacopoeia. New volume was obtained after the 1250 tapping cycles (known as tapped volume) and was subsequently adopted in the determination of *ρ_tap_*. The flowability for each drug–*γ*-CD powder was derived from the *ρ_bulk_* and *ρ_tap_* over the respective Hausner ratio (*H_R_* = *ρ_tap_*/*ρ_bulk_*).

### 2.4. In Vitro Aerosol Performance Determined Using an Andersen Cascade Impactor

HandiHaler, supplied by Boehringer Ingelheim (Ingelheim, Germany), along with an induction port (USP sampling inlet) and Andersen cascade impactor (ACI, TE-20801, Tisch, Cleves, OH, USA), was employed herein for aerosol performance evaluation. Hydroxypropyl methylcellulose capsules (size 3), loaded with 20.0 ± 0.5 mg sample powder, were placed in the designated holder in the HandiHaler. An air stream of 60 L/min was channeled through the holder, with the flow rate precisely controlled by a critical flow controller (TPK 2000, Copley, UK). During the process, the outlet-to-inlet pressure ratio of this critical flow controller was strictly maintained at <0.5 for a stabilized airflow throughout the evaluation. Notably, the aerodynamic cut-off diameter for the first ACI stage (stage 1) was calibrated as 8.6 μm, and was then progressively reduced to 6.5 μm, 4.4 μm, 3.3 μm, 2.0 μm, 1.1 μm, 0.54 μm, and 0.25 μm at stages 2–8, respectively. The *γ*-CD particles and drug–*γ*-CD composites deposited at each stage were assayed by the gravity and HPLC method (following Section 2.5), respectively. The mass difference between the capsule before and after aerosolization (i.e., emitted fillings) was determined as the emitted dose (*ED*), and the *ED* fraction (*ED*, %) was further derived in relation to the total dose (*TD*) used. Significantly, only particles with aerodynamic diameters <5 μm, which were deposited at the 3rd–8th stage of ACI assay, would be counted as fine particle dose (*FPD*). The percentage of *FPD* to *TD* was determined and defined as fine particle fraction (*FPF*, %). To calculate mass median aerodynamic diameter (*MMAD*), the accumulated mass percentage of sample with smaller aerodynamic diameter in respect to ACI cut-off diameter was determined and plotted against the effective cut-off diameter on a log-scale graph [19]. All testing was triplicated (*n* = 3) under similar conditions of 40 ± 5% relative humidity at room temperature. The standard deviations of the results were determined to illustrate the statistical reliability of the experimental data.

### 2.5. HPLC Analysis of BDP

The BDP content in composites was revealed using HPLC (Varian, model 210, Palo Alto, CA, USA) equipped with a Quasar C18 column (5 μm, 150 mm × 4.6 mm) from PerkinElmer. Methanol and water (93:7) were used as the mobile phase and flushed through the column at 1.0 mL/min for compositional separation. For analysis, a 20 μL sample was injected into the column, where the BDP content was subsequently determined from ultraviolet (UV) absorption at 254 nm. Co-existing *γ*-CD and LEU exhibited no absorbance at this wavelength, and therefore would not interfere with the determination of the BDP content. A linear absorbance plot was obtained for BDP content ranging from 0.05 to 50 μg/mL, where the area of absorbance spectrum, which peaked at 254 nm (*y*), can be described by the following equation in respect to the BDP concentration (*x*): *y* = 65.142*x* + 10.459 (*R*^2^ = 0.9999).

### 2.6. Drug Content Determination and In Vitro Dissolution Tests

BDP–*γ*-CD composite microparticles were suspended in ethanol at a loading of 0.25 g/L with a prolonged sonication of 2 h performed to completely dissolve the BDP. Ethanol-treated samples were separated via centrifuging at 10,000 rpm for 10 min, where the supernatant was subjected to HPLC analysis (Varian, model 210, Palo Alto, CA, USA) for content determination after filtering through a 0.45 μm membrane. Significantly, the absorption at 254 nm was the major descriptor of BDP content, which further derived into drug content (%, *w*/*w*) of the composite particles when compared to the weight of pre-dissolved BDP–*γ*-CD composite. All the results were averaged from the triplicated experiments. 

The USP guidelines served as the major guide for the dissolution test in this study, with apparatus 2 (paddle method, DT6, Shin Kwang, Taiwan) employed for the evaluation. Initially, SAA powders containing 5 mg BDP-equivalent were loaded into capsules made of hard gelatin at size 0 prior to the test. These capsules were then subjected to a dissolution test in 1000 mL diluted PBS solution (50 mM, pH = 7.0) at 310.2 ± 0.5 K, which rotated at 50 rpm. A sinker basket was used to ensure the full immersion of the capsule during the test. Aliquot liquid (5 mL) was sampled at suitable time intervals, and equal volume of fresh solvent was immediately added back to retain the testing condition. The BDP dissolved in the liquid sample was analyzed using an HPLC spectrophotometer following method described in Section 2.5. Thereafter, the dissolution profiles of the as-received BDP and composites with different mass ratios (*Z*) between *γ*-CD and BDP were determined to illustrate the drug release properties of the prepared samples.

## 3. Results and Discussion

### 3.1. Solvent Effects on the γ-CD Particles

**Figure 1:**
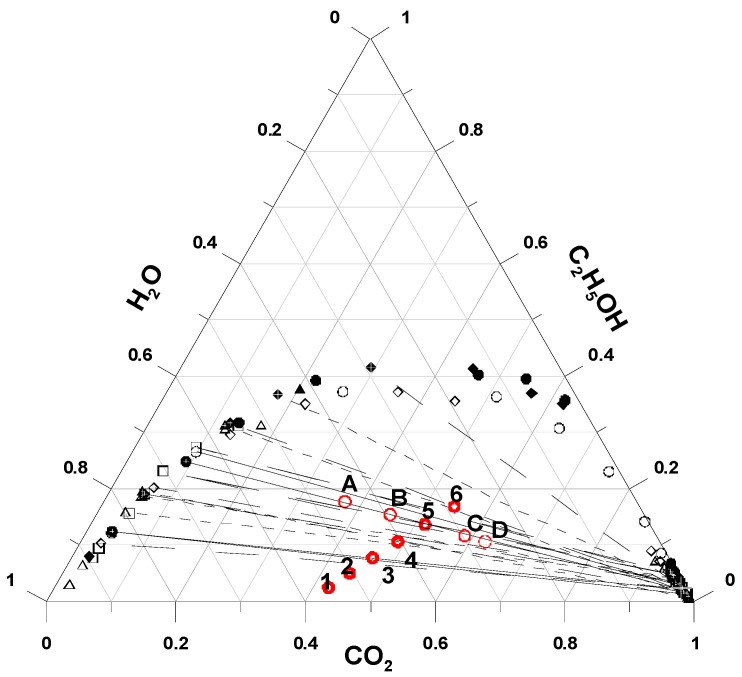
VLE phase diagram for the CO_2_-water-ethanol ternary mixture [25,26]: (●) *T* = 343.2 K, 11.3 MPa; (○) *T* = 343.2 K, 11.8 MPa; (◆) *T* = 333.2 K, 10.1 MPa; (◇) *T* = 333.2 K, 11.8 MPa; (▲) *T* = 313.2 K, 7.9 MPa; (△) *T* = 313.2 K, 10.1 MPa; (☐) *T* = 313.2 K, 10.0 MPa. Symbols of circle (1), (2), (3), (4), (5), and (6) refer to the feed mixture compositions (*R* = *F_CO_*_2_/*F_l_* = 1.8) at ethanol contents of 10%, 20%, 30%, 40%, 50%, and 60% (*w*/*w*), respectively. Symbols (A), (B), (C), and (D) refer to the feed mixture compositions as volume flow ratios of CO_2_ to 50% (*w*/*w*) aqueous ethanol solution (*R* = *F_CO_*_2_/*F_l_*) of 1.0, 1.4, 2.4 and 2.8, respectively.

The CO_2_–water–ethanol vapor-liquid equilibrium (VLE) phase diagram in Figure 1 provides a quick behavior estimation for the ternary mixtures in the saturator. Representatively, the densities of CO_2_ at 276.2 K and 6.5 MPa, and of 50 wt% aqueous ethanol at 298 K and atmospheric pressure, were determined as 934 [20] and 910 kg/m^3^ [21], respectively, with the volumetric flow ratio of CO_2_ to 50 wt% aqueous ethanol solution (*F_CO_*_2_/*F_L_*) of 1.80. From the mass flow perspective, the ratio of CO_2_:aqueous ethanol solution (*m_CO_*_2_/*m_L_*) is known to be 1.85, and therefore falls well into the two-phase region (H_2_O-rich liquid phase and CO_2_-rich vapor phase), as indicated by the circle symbol numbered 5 in Figure 1. The CO_2_ solubility in the ternary liquid system consisting of CO_2_, water, and ethanol (with a mole fraction of approximately 0.10) was quintupled, as compared to that in the CO_2_–water binary system (with a mole fraction of <0.02) [22]. This permits an enhanced atomization of ethanol-containing systems, which is attributed to their relatively lower surface tension and viscosity [23]. Upon forcing the solution through the spray nozzle, the immediate expansion of CO_2_ caused by the instantaneous pressure release resulted in finely atomized droplets, thereby producing fine solid particles during the spray drying process [24]. 

**Figure 2:**
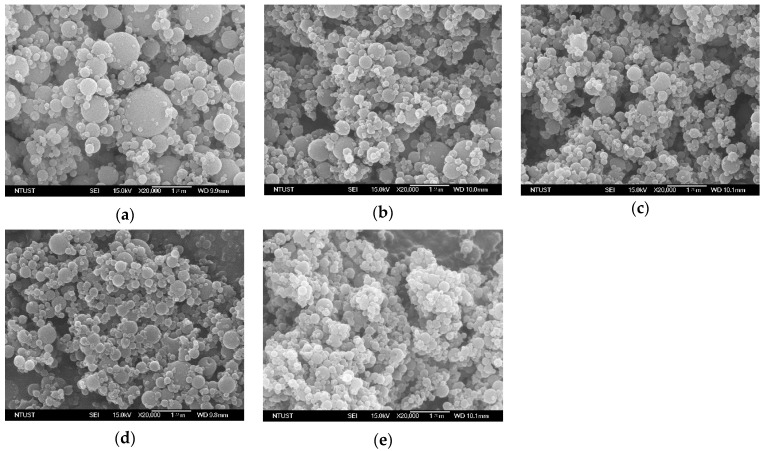
FESEM images of *γ*-CD particles produced by SAA process at different solution ethanol content (%, *w*/*w*) of: (**a**) 0%, (**b**) 30%, (**c**) 40%, (**d**) 50%, (**e**) 60%. (*C_CD_* = 5 mg/mL, *Ts* = 353 K, *T_P_* = 373 K, *R* = 1.8).

**Figure 3:**
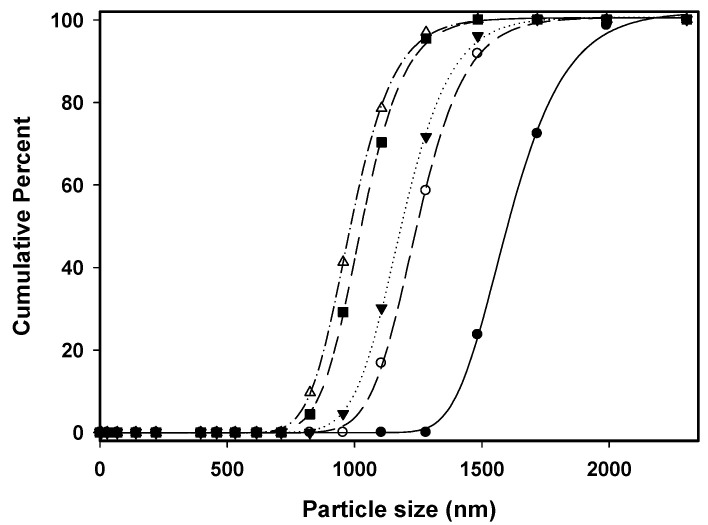
The *PSDs* of the *γ*-CD particles produced by SAA at different solution ethanol contents (%, *w*/*w*): (●) 0%; (○) 30%; (▼) 40%; (■) 50%; (△) 60%.

Table 1 lists the particle size of the SAA-derived *γ*-CD (in terms of *d_no_* and *d*_4,3_), which was prepared using different solutions (with varying ethanol content (EtOH%, *w*/*w*)), *γ*-CD concentration (*C_CD_*), temperatures (precipitator (*T_P_*) and saturator (*T_S_*)), and flow ratio of CO_2_ to *γ*-CD solution (*R*). As mentioned above, increasing ethanol content in *γ*-CD solution could reduce its surface tension and viscosity, thus downsizing the *γ*-CD particles during the SAA process [12,27,28,29,30]. This was further confirmed by the FESEM images shown in Figure 2. Notably, spherical *γ*-CD particles were obtained from the SAA process using water as the sole solvent (Figure 2a, ethanol content = 0%). The increase of ethanol content in the *γ*-CD solution (up to 60%, Figure 2b–e) was found to alter not only the size, but also the morphology of the resultant particles. This can be explained by the increase in the Peclet number (*Pe*), which contributed to the rapid evaporation of ethanol solvent relative to the diffusion of the solute from the surface. Consequently, less spherical *γ*-CD particles were produced despite the maintenance of other parameters [31]. The *PSDs* of the *γ*-CD particles prepared with various ethanol contents are shown in Figure 3. Evidently, *γ*-CD particles were downsized from 1.8 μm to 1.1 μm as the ethanol content in the solution increased from 0 to 60 wt%. Although the presence of ethanol is beneficial for the micronization of particles, the sphericity of the resulting powder, which is directly related to the flowability, is equally important. To obtain sufficiently micronized spherical particles, a solution of 50 wt% aqueous ethanol was used as the solvent, and the effects of the precipitation parameters on the *γ*-CD particle size were investigated.

### 3.2. Effects of the Precipitation Parameters on γ-CD Particle Size

**Figure 4:**
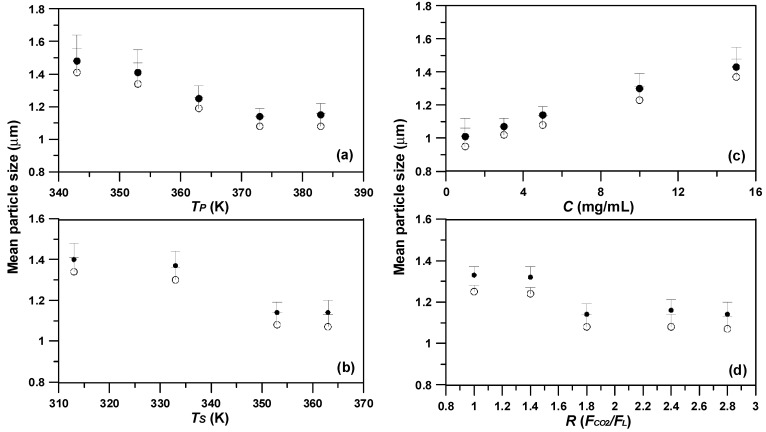
Arithmetic mean size (*d_no_*, ○) and mass-weighted mean particle size (*d*_4,3_, ●) of the *γ*-CD particles varying with SAA process parameters of: (**a**) the temperature of precipitator (*T_P_*), (**b**) the temperature of saturator (*T_S_*), (**c**) the concentration of the *γ*-CD solution (*C_HP_*), (**d**) the volume flow ratio of CO_2_ to *γ*-CD solution liquid (*R*).

SAA were performed using a solution with *C_CD_* of 5 mg/mL and *R* of 1.8 (Table 1, runs #4, #6–12) at varied *T_P_* and *T_S_* to examine the dependence of mean particle size (*d*_4,3_) of the micronized *γ*-CD particle over precipitation parameters. As shown in Figure 4a,b, the *γ*-CD particle size was inversely proportional to both *T_P_* and *T_S_*, plausibly due to the reduced viscosity of the CD solution under high temperatures. This can be confirmed from previous studies that obtained a similar trend against temperatures in the respective SAA processes [22,28,32,33]. Thus, the conducive conditions for producing fine yet narrowly distributed particles were found to be 373.2 K and 353.2 K for the precipitator and saturator temperature, respectively.

Next, the effect of CD concentration (*C_CD_*) in the pre-SAA solution on the *γ*-CD particle size was examined in the range of 1–15 mg/mL (Table 1, runs #4, and #13–16). Figure 4c indicates the direct correspondence of the *γ*-CD particle size with *C_CD_* in the solution, plausibly owing to the increased viscosity of the *γ*-CD solution at high concentrations. This, in turn, results in the formation of large liquid droplets, thereby enlarging the *γ*-CD particle in terms of mean size (*d*_4,3_) during the SAA process. In addition, operation with different flow ratios (*R*) of *F*_CO2_/*F*_L_ could also yield notable effects on the particle size of the resulting *γ*-CD. In experiment runs increasing *R* from 1.0 to 2.8 (Table 1, runs #4, and #17–20), SAA-derived *γ*-CD exhibited a decreasing mean particles size (*d*_4,3_), as depicted in Figure 4d. This can be explained by the presence of moderately excess gas (CO_2_), which provided the energy required for the liquid breakup and fine atomization of an aqueous solution during SAA [34,35]. Previous studies conducted by Reverchon and Antonacci [36], and Wu et al. [22,32] demonstrated the aforementioned phenomenon, thus justifying their similar results in polymer micronization using SAA.

### 3.3. In Vitro Aerosolization Performance of γ-CD Carrier Particles with the Addition of l-Leucine

LEU is an effective dispersant in inhalable drug formulation [6,17,28,37,38]. Thus, the aerosol performance of *γ*-CD carrier particles with LEU added into the formulation is examined herein. Significantly, *γ*-CD particles were pre-synthesized using 5 mg/mL *γ*-CD solution, along with the optimum SAA parameters as follows: precipitator temperature (*T_P_*), 373.2 K; saturator temperature (*T_S_*), 353.2 K; and flow ratio (*R*) of CO_2_ to *γ*-CD solution, 1.8. Six sets of in vitro aerosolization experiments were conducted with varying *C_LEU_*, ranging from 0 to 1.0 mg/mL. The obtained results, including the mean particle sizes (*d*_4,3_) of the *γ*-CD carrier particles (*γ*-CD–LEU), in vitro aerodynamic properties (*FPF* (%), and *MMAD* (μm)), as well as tapped density (*ρ_tap_*) and Hausner ratio (*H_R_* = *ρ_tap_*/*ρ_bulk_*) of the samples, are presented in Table 2.

**Figure 5:**
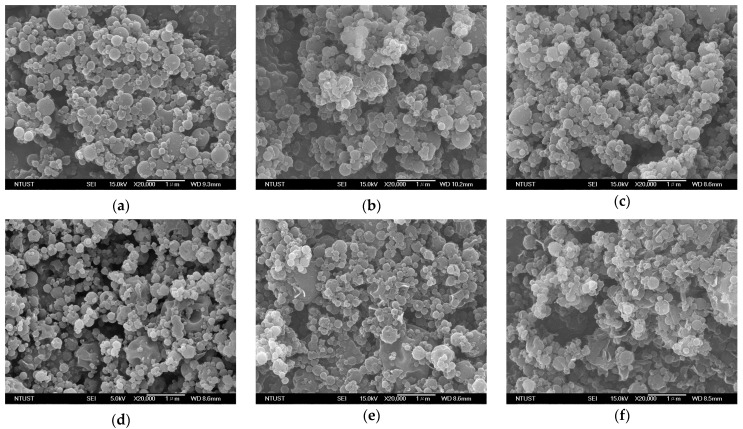
The morphology of SAA-produced *γ*-CD carrier particles with different leucine content: (**a**) 0%, (**b**) 1%, (**c**) 5%, (**d**) 10%, (**e**) 15%, (**f**) 20%.

**Figure 6:**
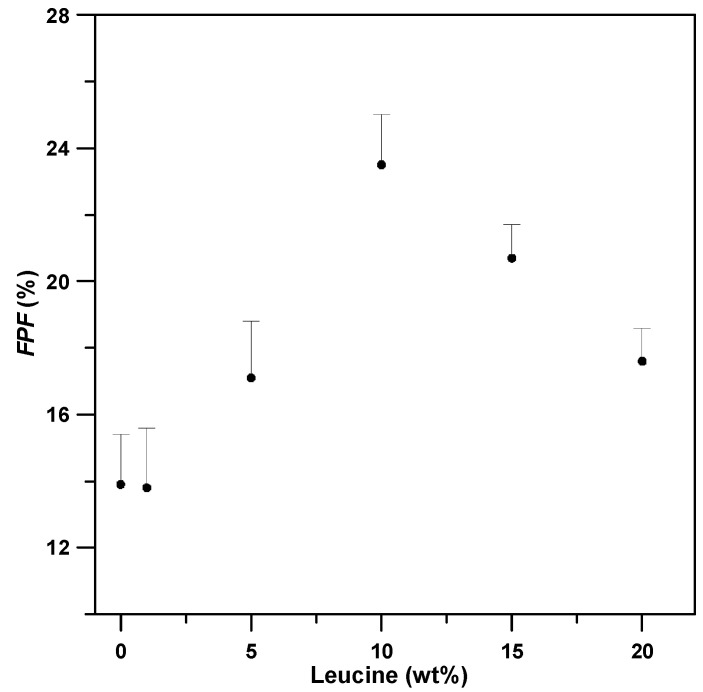
Fine particle fractions (*FPFs*) of *γ*-CD carrier powder varying with the addition of leucine (wt%).

As evidenced by the FESEM images of SAA-*γ*-CD carrier particles (Figure 5), the addition of LEU (0–20 wt%) had an insignificant effect on the particle size of the *γ*-CD carrier particles. However, the sample prepared in the presence of 10% LEU exhibited a few tiny crystals on its surface (Figure 5d). In addition, increased LEU content altered the morphology of the spherical *γ*-CD carrier particles by depositing needle-like fibrous crystals on the surface, thus producing pronounced wrinkles and roughened surfaces. The same phenomenon was observed in previous studies on BDP–mannitol composites [15], HP-*β*-CD particles [28], as well as the complex particles derived from sulfobutylether-*β*-cyclodextrin [9]. In vitro aerodynamic evaluation indicated an extremely high ED fraction (ED, %) which was higher than 97% for all prepared samples. However, the *FPF* values exhibited a positive relationship with the LEU content in the *γ*-CD samples (Figure 6), in conjunction with the negative trend of the recorded *MMAD*. These data collectively justified the role of LEU as a dispersion enhancer in the formulated SAA *γ*-CD samples. Vartiainen et al. [6] reported similar results for dry powder inhalation formulations using corticosteroids, HP-*β*-CD, and LEU. They concluded that samples with wrinkled micro-morphology permitted much lower numbers of inter-particles contact areas, thus enabling better aerosolization performance in the process. Following previous studies [9,10,17,37,38,39], a similar contact-minimization strategy was adopted to reduce the cohesion between particles, thereby imparting enhanced aerosol behavior to the pulmonary drug particles.

In vitro aerosol evaluation can also be performed under a lower Hausner ratio (*H_R_*) to unveil the flowability of the particulate powder [40,41]. Based on the results, *γ*-CD particles which were prepared in the presence of LEU exhibited better flowability (runs #L2–L6) in comparison to that of the sample in run #L1 (performed under high *H_R_* condition). In particular, *γ*-CD-LEU particles with an optimum LEU content of 10% exhibited excellent aerosol performance (run #L4), which is comparable to the results of previous studies [17,28,42]. Correspondingly, the *FPF* of the same *γ*-CD particles was significantly increased to 23.5 ± 1.5% (approximately double that of *γ*-CD particles, which had no LEU; run #L1), along with the lowest recorded *MMAD* of 3.44 ± 0.2 µm. However, as the LEU content increased beyond the optimum point to 15% (run #L5), undesirable agglomerates were observed on the surfaces of the *γ*-CD particles, thus indicating the addition of excess LEU (Figure 5e). Consequently, the powder flowability and aerodynamic behavior were concomitantly suppressed, which explained the declining *FPF* (Figure 6) beyond the optimum point of 10%. 

**Figure 7:**
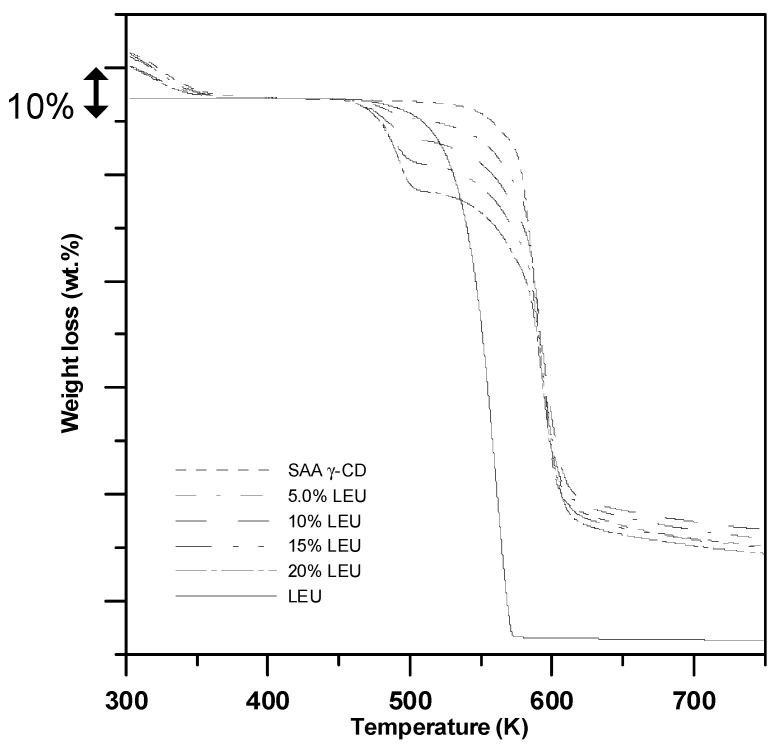
TGA analyses of the *γ*-CD carrier particles with varying amount of LEU (%, *w*/*w*) produced through SAA.

Figure 7 shows the thermogravimetric properties of the SAA-produced *γ*-CD particles with different LEU content. The as-received LEU was subjected to the same thermal evaluation as the blank study. Evidently, one-stage sublimation occurred at 473–573 K, with all samples decomposing into the gas phase (△m = 100%); these results confirmed the low thermal stability of LEU. Similar results were obtained by Li et al. [43], thereby confirming the instability of LEU during heat treatment. Based on this characteristic, the LEU content of each *γ*-CD particle was accurately computed from the weight removed at 573 K. Compared with the bare *γ*-CD particles (Figure 7), the LEU contents of the 5.0, 10, 15, and 20 wt% *γ*-CD–LEU carrier particles were 5.1 ± 0.5, 9.8 ± 0.3, 14.9 ± 0.3, and 19.8 ± 0.4%, respectively, which are highly consistent with the LEU formulations (*C_LEU_*) in Table 2.

### 3.4. In Vitro Aerosolization Performance of the Drug–γ-CD Formulation

The aerosolization performance of fine spherical *γ*-CD particles, prepared with the above determined optimal parameters, was evaluated. Significantly, the results tabulated in Table 3 further justified the function of LEU as a dispersion enhancer in the formulated drug. Upon comparing the performances of BDP–*γ*-CD–LEU with 1% (*C_LEU_* = 0.05 mg/mL, runs #B2) and 10% LEU (*C_LEU_* = 0.5 mg/mL, runs #B3), the latter sample exhibited higher *FPF* (28.25 ± 1.53%) along with lower aerodynamics diameter (*MMAD* = 5.96 μm) and Hausner ratio (*H_R_* = 1.41) compared with those of 1% LEU sample (*FPF* = 15.0 ± 1.5%; *MMAD* = 9.32 μm; *H_R_* = 1.48). 

**Figure 8:**
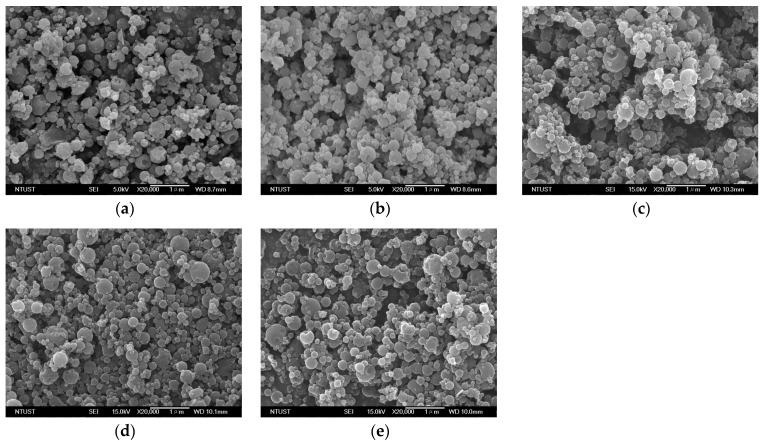
The morphology of drug–*γ*-CD composites obtained from SAA at varied *γ*-CD/BDP mass ratio (*Z*): (**a**) *Z* = 10, (**b**) *Z* = 15, (**c**) *Z* = 20, (**d**) *Z* = 30, (**e**) *Z* = 35.

**Figure 9:**
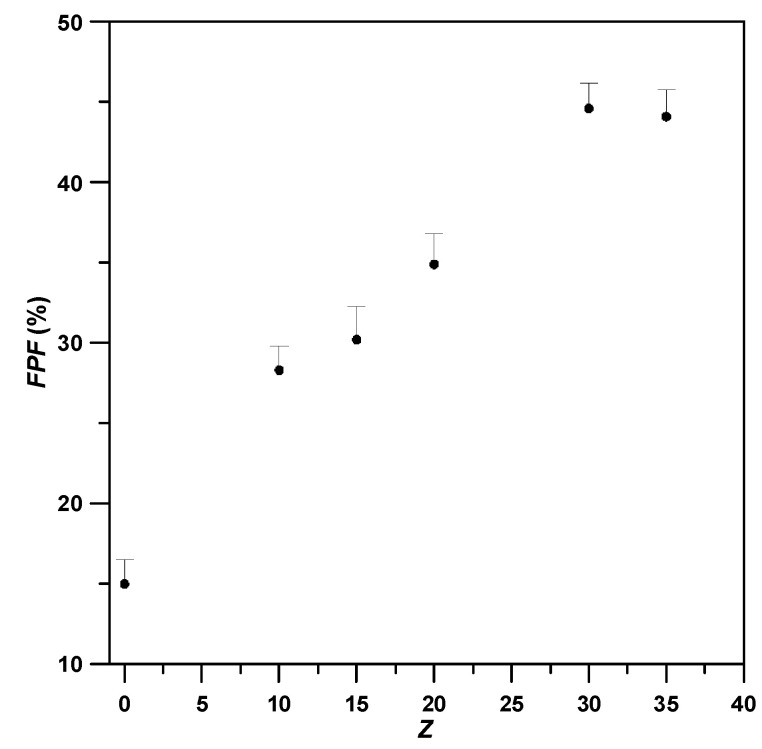
Fine particle fractions (*FPFs*) of drug-carrier composites powder varying with different *γ*-CD/drug mass ration (*Z*).

At a fixed LEU content at 10%, runs #B3–#B7 in Table 3 reveal the correlation between aerodynamic performance of CD/LEU/BDP composites and *γ*-CD-to-drug mass ratio (*Z* = *γ*-CD/BDP). According to FESEM images in Figure 8, the mean particle size of the composite particles does not manifest any obvious changes with the *Z* ratio in the formulation. However, this yielded a considerably high aerodynamic performance for the composite particles, where the *FPF* values increased with *Z* values, as shown in Figure 9. With optimum *Z* of 30, the resultant composite particles exhibited excellent aerodynamic performance, as evidenced from its three-fold increase in *FPF* value (run #B6, 44.56 ± 1.6%) compared with that of the pristine BDP (run #B1, 15.0 ± 1.5%). This is attributed to its small aerodynamic particle size (*MMAD*) and good flowability (low Hausner ratio). Therefore, samples with a high mass ratio (*Z* > 30) are speculated to render a good distribution of BDP in the composite drug particles while promising precise delivery to the deep respiratory system.

### 3.5. In Vitro Dissolution Performance of the Drug–γ-CD Formulation

According to Table 3 (runs #B3–#B7), the good agreement between the experimental and theoretical drug composition of *γ*-CD composite particles at different *Z* ratios indicates high adequacy of SAA in the resulting samples with good solid dispersion. This promises drug composite particles with uniformly dispersed solid solutions, which are critical for the precise formulation of dry powder inhalers.

**Figure 10:**
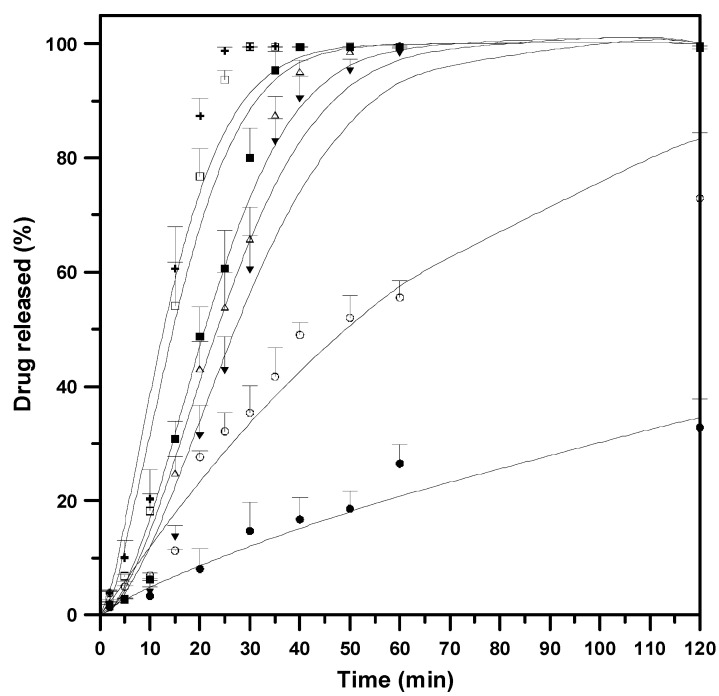
Dissolution profiles of drug-carrier composites powder varying with different *γ*-CD/drug mass ratio (*Z*): (●) unmodified BDP, #B1; (○) physical mixture, *Z_PM_* = 10; (▼) *Z* = 10, #B3; (△) *Z* = 15, #B4; (■) *Z* = 20, #B5; (◻) *Z* = 30, #B6; (**+**) *Z* = 35, #B7.

Currently, a standard in vitro dissolution test for the dry powders used for inhalation has not been proposed. May et al. [44] reported that the paddle method is superior to Franz cell and flow through cell (apparatus 4) because a paddle apparatus with membrane holder can distinguish drugs with slight solubility differences and shows good reproducibility. Therefore, this study adopted the paddle method for the in vitro dissolution test. Specifically, the in vitro evaluation of the dissolution rate of samples with varied *Z* was performed in 50 mM PBS (pH = 7.0) solution. According to Figure 10, the unmodified BDP, with its low solubility, required 36 h for complete drug release. However, this can be augmented by the establishment of drug–*γ*-CD composite particles, as evidenced by their higher drug release rates (Figure 10). In particular, the SAA-derived drug composite particles released >60% of the drug in merely 30 min, whereas the complete release was shortened to only 60 min. This could be ascribed to the synergism offered by the water-soluble *γ*-CD carrier, which effectively improved the dissolution rate of poorly soluble BDP. 

The drug-release kinetics for each sample was evaluated using the Weibull model with the regression parameters tabulated in Table 4. High *r*^2^ values (≥0.91) were obtained for all samples, except those with low drug content (*Z* ≥ 30), thereby confirming the adequacy of the Weibull model for describing drug release behavior. Significantly, the reciprocal of the estimated time for 63.2% drug release (*k_W_*) from the drug–*γ*-CD composites with *Z* = 35 was 20 times that of the as-received BDP, thus suggesting that a higher *Z* sample can prompt an immediate-release inhalation formulation for better drug delivery. In addition, our previous study showed that the solubility of BDP in pure water is 0.28 ± 0.1 mg/L. An aqueous solution containing 5% (*w*/*v*) HP-β-CD increased the solubility of BDP to 37.2 mg/L, which is 133 times that of the intrinsic BDP solubility. Therefore, the dissolution test results of the BDP–HP-β-CD composites and the as-received BDP demonstrated that the reciprocal of the estimated time of 63.2% drug release from BDP–HP-β-CD composites was approximately five times that of the as-received BDP [11]. In this study, a 5% (*w*/*v*) γ-CD solution increased the solubility of BDP in aqueous solution to 93.5 mg/L, thus indicating that the efficiency of γ-CD in improving BDP solubility was better than that of HP-β-CD and accompanied by fast dissolution of BDP–γ-CD formulation.

### 3.6. Characterization of SAA-Derived Drug Composites

**Figure 11:**
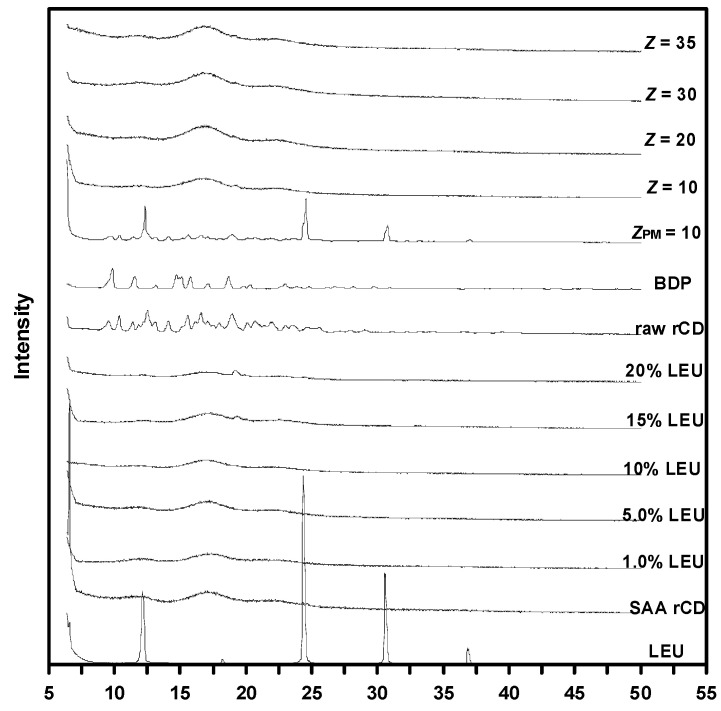
XRD analysis results for *γ*-CD carrier and drug-carrier composites generated over SAA method.

Based on Figure 11, several low-intensity peaks were observed in the as-received *γ*-CD (9.3°, 13.9°, and 18.7°) [45], and the SAA-produced *γ*-CD particles (denoted as SAA *γ*-CD in Figure 11) exhibited three broad peaks (12°, 17°, and 22°), vividly proving the amorphous nature of these samples. The characteristic peaks of crystalline LEU, located at 12.1°, 18.4°, 24.4°, and 30.6° [46], were barely visible in the LEU-added *γ*-CD samples. When the LEU content exceeded 15%, a foreign peak emerged at 20°, which could be related to crystallized LEU on the surface of the carrier particles [9,28,42,47]. This was further confirmed by the FESEM images in Figure 5d–f, where LEU crystals were deposited on the surface of the *γ*-CD carrier particles. The unmodified BDP exhibited a set of characteristic peaks at 9.6°, 11.4°, and 14.9° [48]. Interestingly, the sample with the physical mixing of BDP, LEU (12.1°, 20°, 24.4° and 30.6°), and *γ*-CD (denoted as *Z_PM_* = 10 in Figure 11) exhibited peaks for all constituents; however, most of these peaks were drastically suppressed in the SAA-derived sample with *Z* = 10, despite the same formulation. This could be because of the good dispersion of the constituents in the SAA-produced composites at varied mass ratios (*Z*), which tend to be amorphous.

**Figure 12:**
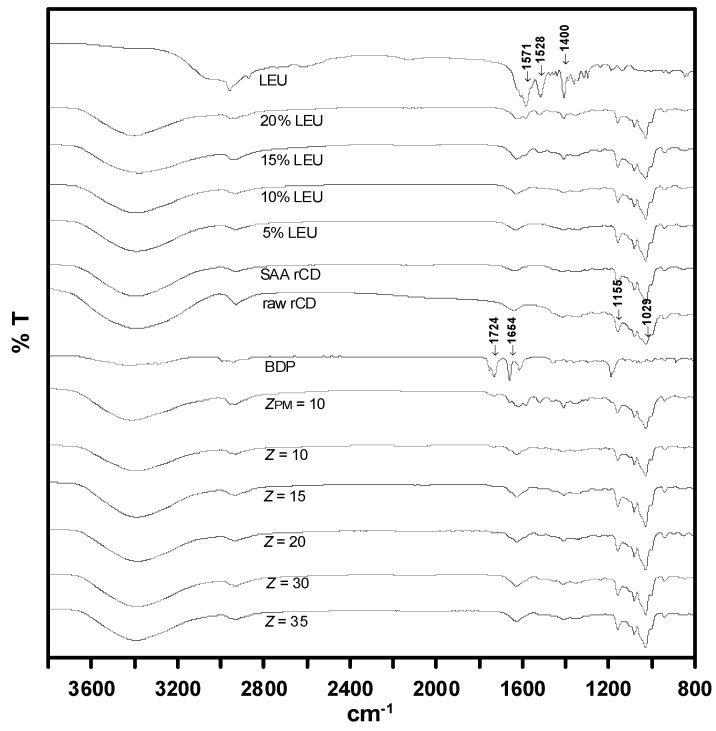
FTIR analysis results for *γ*-CD carrier and drug-carrier composites produced through SAA.

The FTIR spectra presented in Figure 12 constantly displayed absorption spectrums which peaked at 1155 and 1029 cm^−1^, respectively, for all samples. Such absorption bands could be assigned to C–O and C-H groups in *γ*-CD, and could therefore emerge in all the measured samples. On the other hand, absorption related to LEU can be observed at 1571 and 1400 cm^−1^, which signify the presence of asymmetric and symmetric stretching modes of COO^–^ ion group in the composite. Meanwhile, the peak at 1528 cm^−1^ could be prompted by the N–H^+^ stretching upon excitation during IR scanning. As for BDP, the stretching activities of its conjugated and non-conjugated C=O can be traced from the bands peaked at 1724 and 1654 cm^−1^, respectively. Interestingly, the characteristic bands of *γ*-CD, LEU and BDP were only observable in the spectrum of the physically mixed sample (*Z_PM_* = 10), whereby those of BDP are found absence in the drug–*γ*-CD composite particles. This could be attributed to how the C=O stretching bands of BDP were restricted by inclusion within the cavity of *γ*-CD.

**Figure 13:**
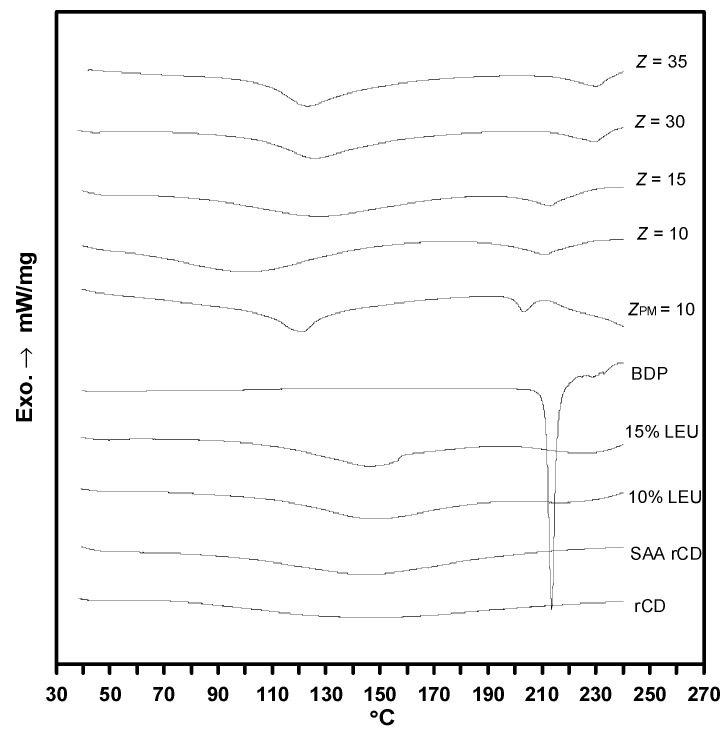
DSC results for *γ*-CD carrier and SAA-derived drug-carrier composites.

Significantly, the DSC results of the SAA-derived drug-carrier composites samples were presented in Figure 13. The embedment of the guest molecule (drug) in the cyclodextrin cavity or the formation of an inclusion complex causes the disappearance of or shift in the melting point of the guest drug [49]. Based on the results, the BDP drug undergoes melting at 213 °C, whereas *γ*-CD in amorphous phase exhibits a broad endothermic peak from 50–150 °C, plausibly arisen from its dehydration process. Similar broad dehydration endothermic peak of *γ*-CD was inherited by the physically mixed sample in conjunction to the slight reduction of BDP melting point to 205 °C. This can be explained by the partial interaction between BDP and *γ*-CD during physical mixing, which reduces the crystallinity of the drug. This is not uncommon, as a similar melting point reduction was also observed in the *β*-CD/salbutamol composites produced by the physical mixing method [50]. With the same mass ratio of *Z* = 10, BDP melting peak almost disappeared in the SAA-derived BDP–*γ*-CD composites compared with the physically mixed sample (*Z_PM_* = 10). This could be related to high amorphous phase in the sample, indicated by the XRD patterns in Figure 11. An increase in *Z* value rendered the samples more amorphous, thereby further enhancing the dissolution rate of BDP during drug delivery. This is supported by the results obtained in the dissolution experiment.

## 4. Conclusions

Inhalable drug-excipient composite particles consisting of BDP and *γ*-CD were successfully produced using single-step SAA process. Results indicate that 50% (*w*/*w*) ethanolic solvent could yield fine spherical particles with excellent aerosolization performance. LEU appeared to be an effective dispersion enhancer for active ingredient, with optimum composition determined as 10 wt%. Meanwhile, experiments with varied *γ*-CD-to-BDP (*Z*) mass ratio confirmed that BDP–*γ*-CD composite particles derived from high *Z* of 30 (run #B6) exhibited the best aerodynamic performance, with a three-fold *FPF* value of 45% (run #B6) recorded against the unmodified BDP (run #B1, 15%). The dissolution experiment, on the other hand, revealed the adequacy of water-soluble excipient (*γ*-CD) in facilitating the dissolution rate of BDP, significantly reducing the complete dissolution time from 36 h to merely 60 min. This study unveils the potential of SAA in preparing not only BDP–*γ*-CD drug composites, but also any instant dry powder formulations that aim to improve the bioavailability of inhaled drugs with solubilities [51].

## Figures and Tables

**Table 1:** The mean particle size of SAA-derived *γ*-CD particles prepared over different experimental conditions.

Run	EtOH	*T_P_*	*T_S_*	*C_CD_*	*R*	*d_no_*	*d*_4,3_
	wt%	K	K	mg/mL		μm	μm
1	0	373	353	5	1.8	1.74 ± 0.15	1.80 ± 0.14
2	30	373	353	5	1.8	1.35 ± 0.07	1.42 ± 0.06
3	40	373	353	5	1.8	1.29 ± 0.03	1.36 ± 0.03
4	50	373	353	5	1.8	1.12 ± 0.10	1.18 ± 0.10
5	60	373	353	5	1.8	1.08 ± 0.06	1.14 ± 0.05
6	50	343	353	5	1.8	1.41 ± 0.15	1.48 ± 0.16
7	50	353	353	5	1.8	1.34 ± 0.13	1.41 ± 0.14
8	50	363	353	5	1.8	1.19 ± 0.07	1.25 ± 0.08
9	50	383	353	5	1.8	1.08 ± 0.08	1.15 ± 0.07
10	50	373	313	5	1.8	1.34 ± 0.07	1.40 ± 0.08
11	50	373	333	5	1.8	1.30 ± 0.06	1.37 ± 0.07
12	50	373	363	5	1.8	1.07 ± 0.06	1.14 ± 0.06
13	50	373	353	1	1.8	0.95 ± 0.11	1.01 ± 0.11
14	50	373	353	3	1.8	1.02 ± 0.05	1.07 ± 0.05
15	50	373	353	10	1.8	1.23 ± 0.08	1.30 ± 0.09
16	50	373	353	15	1.8	1.37 ± 0.11	1.43 ± 0.12
17	50	373	353	5	1.0	1.25 ± 0.03	1.33 ± 0.04
18	50	373	353	5	1.4	1.24 ± 0.03	1.32 ± 0.05
19	50	373	353	5	2.4	1.08 ± 0.06	1.16 ± 0.05
20	50	373	353	5	2.8	1.07 ± 0.06	1.14 ± 0.06

**Table 2:** Results obtained from the in vitro aerosolization evaluation of *γ*-CD particles prepared in the presence of 0–20 wt% leucine.

Run	*C_LEU_*	*ED*	*FPF*	*MMAD*	*d*_4,3_	*ρ* *_tap_*	*H_R_*
	mg/mL	%	%	µm	µm	g/cm^3^	* ρ _tap_* / * ρ _bulk_*
L1	0	97.6 ± 0.8	13.8 ± 1.8	10.8 ± 0.31	1.14 ± 0.04	0.27 ± 0.02	1.40 ± 0.02
L2	0.05	99.2 ± 0.4	13.9 ± 1.5	8.76 ± 0.32	1.16 ± 0.05	0.26 ± 0.03	1.32 ± 0.01
L3	0.25	99.5 ± 0.2	17.1 ± 1.7	4.54 ± 0.53	1.20 ± 0.05	0.21 ± 0.02	1.31 ± 0.03
L4	0.50	97.5 ± 0.3	23.5 ± 1.5	3.44 ± 0.24	1.25 ± 0.05	0.30 ± 0.02	1.20 ± 0.02
L5	0.75	99.0 ± 0.4	20.7 ± 1.0	4.53 ± 0.31	1.29 ± 0.08	0.22 ± 0.03	1.23 ± 0.01
L6	1.0	98.3 ± 0.3	17.6 ± 1.0	6.28 ± 0.52	1.32 ± 0.09	0.25 ± 0.03	1.24 ± 0.02

**Table 3:** Experimental results of BDP–*γ*-CD composites with different mass ratio (*Z*) produced using SAA.

Run	*C_BDP_*	*Z*	*C_LEU_*	*FPF*	*MMAD*	Drug Cont.	*d*_4,3_	*ρ* *_tap_*	*H_R_*
	mg/mL	-	mg/mL	%	µm	wt%	µm	g/cm^3^	*ρ_tap_*/*ρ_bulk_*
B1 ^a^	-	-	-	17.3 ± 3.0	14.7 ± 0.40	-	-	-	-
B2	0.45	10	0.05	15.0 ± 1.5	9.32 ± 0.14	9.00 ± 0.2	1.11 ± 0.04	0.26 ± 0.02	1.48 ± 0.03
B3	0.41	10	0.5	28.3 ± 1.5	5.96 ± 0.23	8.18 ± 0.2	1.19 ± 0.02	0.26 ± 0.03	1.41 ± 0.02
B4	0.28	15	0.5	30.2 ± 2.1	4.61 ± 0.18	5.62 ± 0.2	1.20 ± 0.03	0.24 ± 0.02	1.41 ± 0.01
B5	0.21	20	0.5	34.9 ± 1.9	4.36 ± 0.24	4.28 ± 0.1	1.24 ± 0.04	0.23 ± 0.01	1.41 ± 0.03
B6	0.15	30	0.5	44.6 ± 1.6	4.36 ± 0.29	2.90 ± 0.1	1.27 ± 0.03	0.22 ± 0.02	1.29 ± 0.02
B7	0.13	35	0.5	44.1 ± 1.7	3.07 ± 0.43	2.50 ± 0.1	1.28 ± 0.03	0.24 ± 0.02	1.33 ± 0.01

^a^ B1: as-received BDP.

**Table 4:** Correlated results of the in vitro dissolution profiles of drug–*γ*-CD composite particles with different mass ratio (*Z*) produced using supercritical assisted atomization.

Run	*Z*	Weibull ^a^			
		*a*	*b*	*r* ^2^	*k_W_* ^b^
B1 ^c^	-	147.3	0.864	0.98	0.0031
B3	10	393.4	1.701	0.92	0.0298
B4	15	351.9	1.743	0.91	0.0346
B5	20	329.7	1.784	0.91	0.0388
B6	30	101.6	1.585	0.89	0.0542
B7	35	60.73	1.470	0.87	0.0613

^a^ Weibull model: $m=1-\exp\left[\frac{-t^{b}}{a}\right]$. ^b^ The reciprocal of the estimated time (min^−1^) of 63.2% drug released from Weibull model, $k_{W}={\left(a\right)}^{\frac{-1}{b}}$. ^c^ unmodified BDP.

## Data Availability

Not applicable.
